# Application of Box-Behnken designs in parameters optimization of differential pulse anodic stripping voltammetry for lead(II) determination in two electrolytes

**DOI:** 10.1038/s41598-017-03030-2

**Published:** 2017-06-05

**Authors:** Xiao-Lan Yu, Yong He

**Affiliations:** College of Biosystems Engineering and Food Science, Zhejiang University, Hangzhou, 310058 P.R. China

## Abstract

Box-Behnken design was advantageous to parameters optimization of differential pulse anodic stripping voltammetry (DPASV) for the analysis of lead(II) with its high efficiency and accuracy. Five Box-Behnken designs were designed and conducted in the electrolyte of 0.1 mol/L acetate buffer and 0.1 mol/L HCl without the removal of oxygen. Significant parameters and interactions in each electrolyte were found (*P*-value < 0.05) and their quantitative effects on lead(II) determination were classified into two categories, linear and quadratic. Though significant parameters and interactions were not similar in different kinds of electrolytes, characteristic parameters of differential pulse voltammetry, which were pulse amplitude, pulse width and interval time, were found significant in both electrolytes. After optimization, peak currents and relative standard deviation at 20 μg/L along with detection limits in both electrolytes were superior than before. With the lower detection limit and R.S.D., 0.1 mol/L HCl was a better choice for electrolytes in this work. Meanwhile, with the combination of parameters optimization and background subtraction, the interference of dissolved oxygen for lead(II) determination was eliminated. It was important and necessary to apply Box-Behnken designs in parameters optimization of DPASV for lead(II) determination regardless of the electrolyte kinds.

## Introduction

Lead, one of the primary heavy metal pollutants, as well as one of the 10 chemicals of major public health concern proposed by World Health Organization (WHO)^1^, is a cumulative toxicant that affects multiple body systems^2^ and exists in nature at a relatively low level of which the widespread occurrence in the environment is largely the result of human activity^3^. Classic analytical methods, including atomic absorption spectroscopy (AAS), inductively coupled plasma optical emission spectrometry (ICP-OES), and inductively coupled plasma mass spectrometry (ICP-MS), perform satisfactorily and reliably in determining lead whether in solid samples or liquid samples. However, complicated procedures in sample treatments, expensive instruments and non-friendly to inexperienced researchers restrict their applications and it’s to some extent tough to determine lead simply, rapidly and on the spot using these analytical methods.

With their low cost and high sensitivity, electrochemical analytical methods, for example, differential pulse anodic stripping voltammetry (DPASV) and square wave anodic stripping voltammetry (SWASV) with detection concentrations ranging from 10^−6^–10^−11^ mol/L^4^, are considered to be promising methods for determining heavy metals besides classic analytical methods. Through preconcentrating the metal M in Hg by the reduction of its ion M^+n^ at a constant potential in the first step, and measuring the reoxydation peak in the second step, along with substituting modulations for lineal scan^4^, DPASV has been applied in lots of areas^4–6^. While, at the same time, various experimental parameters of DPASV, such as the kind of electrolyte, the pH of electrolyte, electrodeposition potential, electrodeposition time, balance time, step increment, pulse amplitude, pulse width and interval time are required to be optimized to get the higher sensitivity for the analysis of lead(II). In the meantime, the interference of dissolved oxygen in the electrolyte needs consideration and optimization.

One-variable-at-a-time is the most common way to optimize experimental parameters in DPASV measurements^5, 7–9^ at present, whereas, it loses sight of the potential interactions between parameters and takes a great deal of time to carry out the whole optimization because it is conducted by monitoring the influence of one parameter on an experimental response at a time with other parameters kept at a constant level^10, 11^. Optimizations using multivariate statistic techniques, which are full three-level factorial designs, Box-Behnken designs, central composite designs and Doehlert designs, overcome these problems^11^. Applied in the optimization of spectroanalytical methods^12, 13^, chromatographic methods^14, 15^, capillary electrophoresis^16, 17^ and sorption process^18, 19^, Box-Behnken designs have attracted wide attention of researchers. Permitting estimations of the parameters of the quadratic model and building of sequential designs, Box-Behnken design, a response surface methodology, is slightly more efficient than the central composite design but much more efficient than the three-level full factorial designs^20^. Notwithstanding, Box-Behnken designs have not been widely used for the optimization of electroanalytical methods. To the best of our knowledge, there is no information available on the application of Box-Behnken designs for parameters optimization of DPASV to analyze lead(II) in two electrolytes currently.

To explore the potential interactions between experimental parameters and to figure out the better choice for the electrolyte, five Box-Behnken designs were developed and conducted for the parameters optimization in lead(II) determination by DPASV without the removal of oxygen in two kinds of electrolytes, and quantitative effects on lead(II) determination of significant parameters and interactions were discussed. Comparisons of peak currents, relative standard deviation (R.S.D.) and detection limits before and after parameters optimization in two electrolytes, advantages of Box-Behnken designs and the interference of dissolved oxygen were also presented.

## Results and Discussion

### Significant parameters and interactions

For Box-Behnken designs carried out in 0.1 mol/L HAc-NaAc and 0.1 mol/L HCl, significant parameters and interactions as well as their quantitative effects and optimal values were demonstrated in Table 1. It was obvious that characteristic parameters of differential pulse voltammetry (DPV), including pulse amplitude, pulse width and interval time, through which a DPV was distinct from the other, whether in single or in interaction, affected the peak current significantly in both electrolytes.

**Table 1:** Quantitative effects of significant parameters or interactions and their optimal values.

Electrolytes	Parameters/Interactions	Quantitative effects	Optimal values
0.1 mol/L acetate buffer (HAc-NaAc)	pH of electrolyte	quadratic	4.45
	pH × pH		
	balance time (s)	linear	30
	pulse amplitude (V)	linear	0.08
	pulse width (s)	quadratic	0.05
	interval time (s) × interval time (s)	quadratic	0.1
0.1 mol/L HCl	electrodeposition time (s)	linear	180
	step increment (V)	linear	0.002
	pulse amplitude (V) × pulse width (s)	quadratic	0.06 × 0.6
	interval time (s) × interval time (s)	quadratic	0.05

### 0.1 mol/L HAc-NaAc

**Figure 1:**
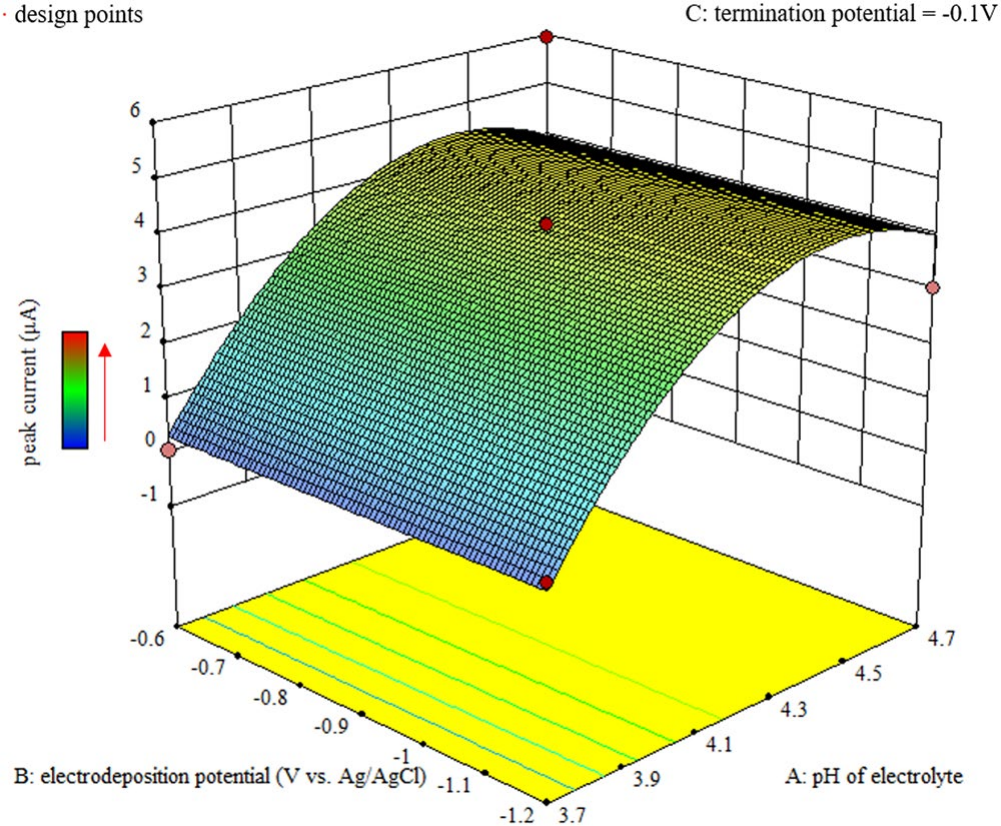
Response surface diagram for pH of electrolyte and electrodeposition potential in 0.1 mol/L acetate buffer.

**Figure 2:**
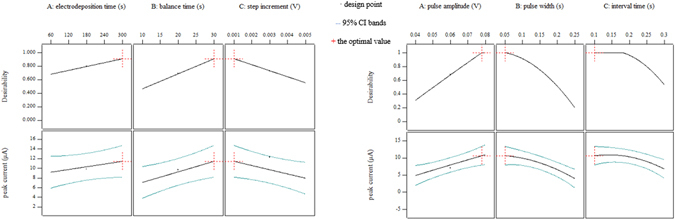
Desirability and 95% prediction intervals of electrodeposition time, balance time, step increment, pulse amplitude, pulse width and interval time in 0.1 mol/L acetate buffer.

Parameters, the pH of electrolyte, balance time, pulse amplitude and pulse width, along with interactions, pH × pH and interval time × interval time were found significant (*P*-value < 0.05) in the electrolyte of 0.1 mol/L acetate buffer, and their quantitative effects were illustrated in Figs 1 and 2.

In the electrolyte of 0.1 mol/L acetate buffer, it had been noted that the pH of electrolyte took profound effects on the stripping currents of target metal ions whether the form of the working electrodes^7, 8, 21^ and the trend of the pH was similar within the range of observation. An increment was in the initial part of the trend with a decrease afterwards. This trend could be contributed to the hydrogen evolution at a more negative potential^22^ and the incomplete reduction of the working electrode at a more positive potential.

The significant influence of balance time, the intermediate process between electrodeposition and determination, on the peak current might be assigned to the difficulty to achieve the balance state when it was too short.

Since pulse amplitude, pulse width and interval time were three characteristic parameters of DPV, they impacted the peak current significantly in their own way. Pulse amplitude: the distance between the bottom and the top of the pulse; pulse width: the length of time of the pulse; interval time: the length of time between two pulses; although their influence mechanisms were not clear enough at present, they were need to be optimized to acquire a higher response.

### 0.1 mol/L HCl

**Figure 3:**
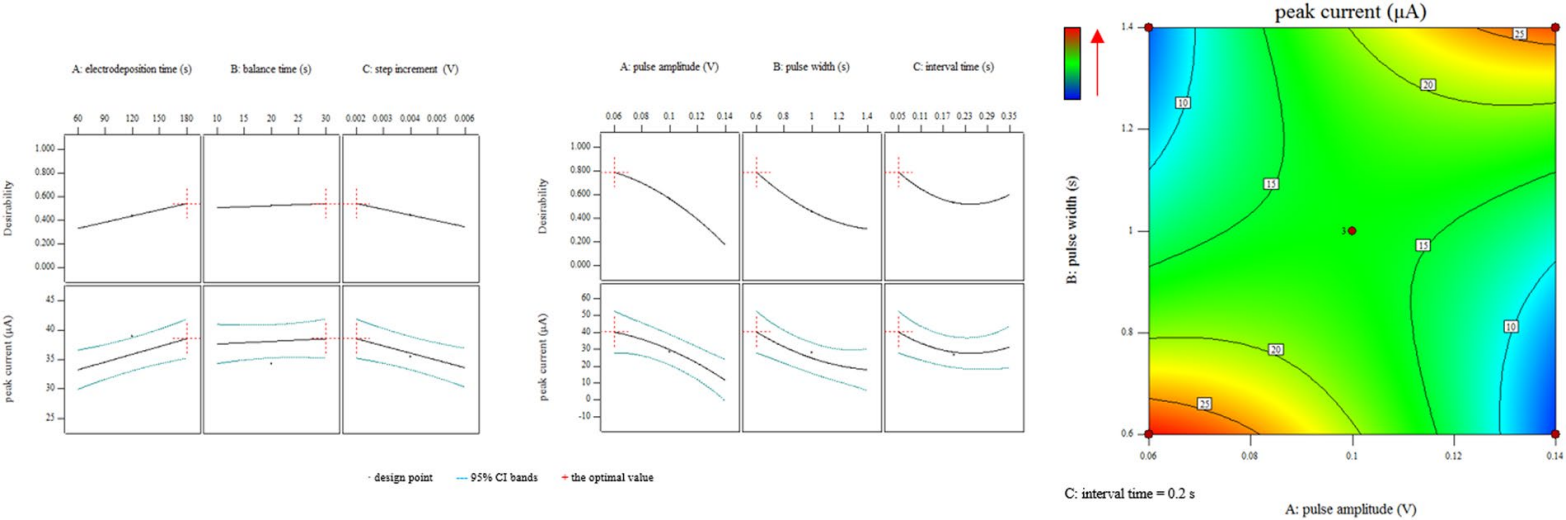
Desirability and 95% prediction intervals of electrodeposition time, balance time, step increment, pulse amplitude, pulse width, interval time and pulse amplitude × pulse width in 0.1 mol/L HCl.

Similar with 0.1 mol/L acetate buffer, the interaction interval time × interval time was also found significant (*P*-value < 0.05) in the electrolyte of 0.1 mol/L HCl, while other significant parameters and interactions were different, which were electrodeposition time, step increment, and pulse amplitude × pulse width. Quantitative effects and interactions of optimized parameters in the electrolyte of 0.1 mol/L HCl were shown in Fig. 3.

In Box-Behnken design I in 0.1 mol/L acetate buffer, except the pH of electrolyte, another two parameters which were electrodeposition potential and termination potential, were not found significant and the pH of 0.1 mol/L HCl was consistent as well as close to the digestion solution, therefore, they were not optimized in the electrolyte of 0.1 mol/L HCl. Values of electrodeposition potential and termination potential were selected as −0.55 V vs. Ag/AgCl and −0.25 V vs. Ag/AgCl by referring to the existing literatures.

It was clear that with the increase of electrodeposition time in a certain range, the amount of metal ions at the working electrode surface greatly increased because of the electrochemical deposition^7^, while the significance of electrodeposition time also depended on the kinds of electrolytes and other parameters.

The smaller the step increment, the denser the dots in the figure produced by DPASV, in which the shape of the peak along with its details was more clear and thus, step increment had significant effects on the peak current in the electrolyte of 0.1 mol/L HCl.

Significant influences of pulse amplitude, pulse width and interval time on the peak current were attributed to their unique characteristics impacting the measurement of lead(II) by DPASV. Their effects might change forms, for example, in single or in interaction, as the kind of electrolytes changed, however, they were required to be optimized before the formal measurement to maximize the peak current for improving the sensitivity of lead(II) determination. Nevertheless, as far as we’ve seen, there is no research aimed at exploring the interactions between these three parameters nor acquiring the optimal values simultaneously at present.

### Quantitative effects of experimental parameters and interactions

Presented in Table 1, quantitative effects of significant parameters and interactions on lead(II) determination through DPASV could be classified into two categories, linear and quadratic.

### Linear

Whether the parameter was significant or not, when its quantitative effect was linear, the parameter would get its optimum at the endpoint within the range of observation in experiments.

In the electrolyte of 0.1 mol/L acetate buffer, balance time and pulse amplitude, along with the non-significant parameter, electrodeposition time, got the optimum at the maximum values as illustrated in Fig. 2 and their optimum values were 30 s, 0.08 V and 300 s, respectively; while, electrodeposition potential, termination potential and step increment, which were not found significant, were going to obtain the optimum at the minimum values with values of −1.2 V vs. Ag/AgCl, −0.2 V vs. Ag/AgCl and 0.001 V, respectively.

In the electrolyte of 0.1 mol/L HCl, though the significance of electrodeposition time, balance time and step increment reversed, their quantitative effects were consistent and their optimum values were 180 s, 30 s and 0.002 V. When it referred to the pulse amplitude, it was obvious that pulse amplitude was significant all the same, however, its quantitative effect was changed into quadratic, just as Fig. 3 demonstrated.

### Quadratic

The optimum value of one parameter when its quantitative effect was quadratic had two choices. One was similar with the linear effects, where one of the endpoint was the optimum. The other was different, that is, the optimum fell between the two endpoints. In our experiments, both cases occurred.

#### Endpoint optimum

Curves of pulse width and interval time in the electrolyte of 0.1 mol/L acetate buffer, as well as pulse amplitude, pulse width and interval time in the electrolyte of 0.1 mol/L HCl, would obtain the maximum response at the endpoint of the range of parameters as Figs 2 and 3 illustrated and their optimal values were given in Table 1.

Figure 3 also showed the interaction of pulse amplitude (V) × pulse width (s). From Fig. 3, it was no difficulty to figure out that when pulse amplitude and pulse width become larger or smaller at the same time, the peak current was higher. The maximum value of the peak current was acquired when the values of pulse amplitude and pulse width were 0.06 V and 0.6 s.

#### Non-endpoint optimum

As for the pH of electrolyte in 0.1 mol/L acetate buffer, whose curve was presented in Fig. 1, the optimal value was 4.45 after the calculation. This result was relatively consistent with current studies where the optimal value of pH for lead(II) determination by DPASV were 4.7^7^, 4.5^8^ and 4.0^23^.

### Peak currents, relative standard deviation and detection limits

The feasibility and necessity of applying Box-Behnken designs for parameters optimization of DPASV in lead(II) determination was confirmed through comparing the peak current, R.S.D. and the detection limit before and after optimization in two electrolytes. Comparisons were given in Table 2 and detection limit (3 σ) was calculated according to IUPAC^24^ recommendations.

**Table 2:** Comparisons of peak currents, relative standard deviation (R.S.D.) and detection limits before and after parameters optimization in two electrolytes.

Optimization	Electrolytes	Peak currents at 20 μg/L (μA)	R.S.D. at 20 μg/L (%)	Detection limits (μg/L)
before	HAc-NaAc	3.79	9.58	5.09
	HCl	33.77	0.61	3.89
after	HAc-NaAc	7.56	1.85	2.27
	HCl	48.39	1.08	0.66

After optimization, peak currents at 20 μg/L increased 99.74% and 43.29% in the electrolytes of 0.1 mol/L acetate buffer and 0.1 mol/L HCl; at the same time, detection limits decreased 55.40% and 83.03%, respectively.

**Figure 4:**
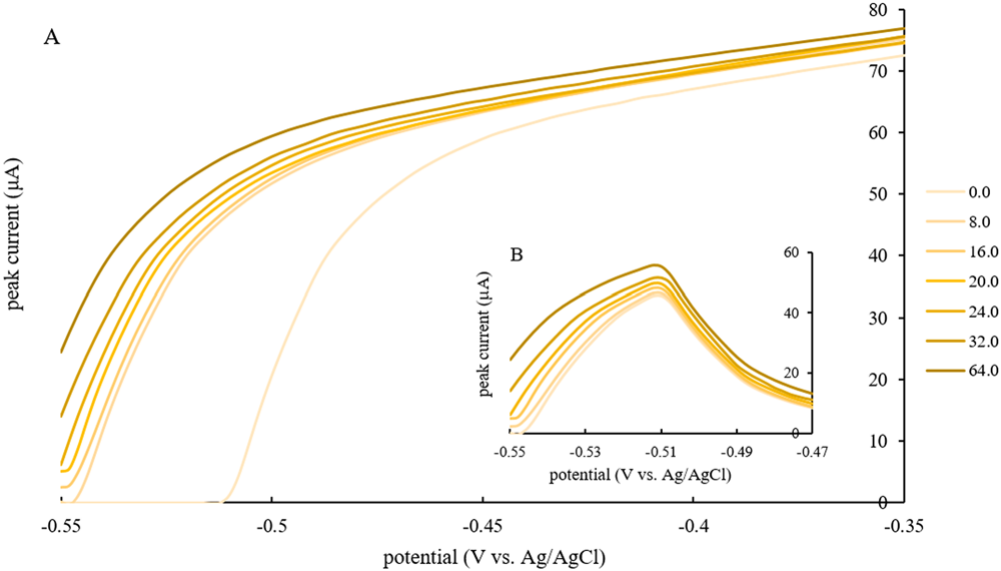
(**A**) DPASV determination on lead(II) without the removal of oxygen in 0.1 mol/L HCl; (**B**) Curves from part (**A**) after background subtraction.

From the perspective of peak currents, R.S.D. and detection limits, 0.1 mol/L HCl was a better choice for the kind of electrolytes in comparison with 0.1 mol/L acetate buffer in this work, and on the basis of IUPAC^24^, the quantification limit (10 σ) in 0.1 mol/L HCl was 2.18 μg/L. The peak current, which was the response, was linear in the electrolyte of 0.1 mol/L HCl in concentrations ranging from 8.0 to 64.0 μg/L (*R* ^2^ = 0.9944) for lead(II) determination. The peak current of each concentration was acquired by background subtraction and curves of DPASV determination on lead(II) without the removal of oxygen in 0.1 mol/L HCl were illustrated in Fig. 4.

### Advantages of Box-Behnken designs

Compared with the contemporary optimization method utilized in DPASV, Box-Behnken designs bear the main advantages of figuring out the potential interactions between parameters and also time-saving by reducing the number of experiments. Significant interactions of pulse amplitude × pulse width and interval time × interval time in the electrolyte of 0.1 mol/L HCl as well as pH × pH and interval time × interval time in the electrolyte of 0.1 mol/L acetate buffer were discovered. Demonstrated in Table 3, the average number of experiments to optimize one factor using one-variable-at-a-time was 6.9, while for Box-Behnken designs, the average number was 5.

**Table 3:** Comparisons between one-variable-at-a-time and Box-Behnken designs in parameters optimization of DPASV for lead(II) determination.

Optimization methods	The number of experiments	The number of optimized factors	Reference
one-variable-at-a-time	16	3	5
	20	3	7
	20	3	8
	11	2	25
	21	3	31
	28	3	28
	18	2	29
	5	1	30
	6	1	35
	14	2	32
Box-Behnken designs	15	3	this work

Saving time for carrying out optimization experiments provided the opportunity to optimize more parameters, such as pulse amplitude, pulse width and interval time in DPASV. These three parameters, or factors, were generally given in researches without the optimization, whereas, they affected the peak current significantly as this work presented. Thus, it was of necessity and importance to optimize these parameters before using DPASV to conduct quantitative analyses for heavy metals, especially when they were in trace amounts.

### Oxygen

The presence of dissolved oxygen in the solution might affect the determination of lead(II) as the peak of lead(II) is situated within the oxygen reduction wave (~−0.2 V)^25^. Nevertheless, a number of researches^25–30^, which employed novel electrodes like nafion-coated bismuth film electrode and gold microwire electrode as the working electrode, declared that the removal of oxygen was not necessary because (i) oxygen was depleted at the electrode during the preconcentration step and (ii) oxygen from the bulk of the solution did not reach the electrode during the short duration of the stripping step^29^.

To improve the sensitivity of determining trace amounts of lead(II) is the purpose for removing dissolved oxygen in the solution. Although Box-Behnken designs in this work were carried out in the presence of oxygen, peak currents acquired were much higher than data from studies where the corresponding concentrations of lead(II) were similar, for example, with the same concentration of lead(II) (20 μg/L) and the same electrolyte (0.1 mol/L acetate buffer), the highest peak current in this work was 12.356 while in ref. 5, the highest peak current was about 0.8. This result evidenced that parameters optimization could alleviate the side effect of dissolved oxygen in the solution on lead(II) determination by DPASV to some extent. Consequently, the measurement time would be decreased based on this finding and on-site or on-line applications by DPASV might be feasible.

On the other hand, the oxygen interference for lead(II) could be eliminated by background subtraction^9, 25^ and background subtraction was applied in this work. Whereas, values fluctuations of peak currents in three Box-Behnken designs conducted in 0.1 mol/L acetate buffer occurred. Frankly speaking, fluctuations of the response were not rare in parameters optimization^8, 22, 25, 28, 31^ since (i) the impact of each parameter was not identical and (ii) the sequence of parameters optimized might have an influence on the response. As for the electrolyte of 0.1 mol/L HCl in this work, without the removal of oxygen in the solution, the peak current at 20 μg/L, R.S.D. at 20 μg/L and the detection limit were all close to, or even better than the corresponding data^5, 7, 26, 27, 32, 33^ in existing researches which removed oxygen from the solutions.

## Conclusions

Box-Behnken designs enjoyed the advantages of efficiency and accuracy in parameters optimization of DPASV for lead(II) determination. Significant parameters and interactions changed as the kind of electrolytes changed, while characteristic parameters of DPV, which were pulse amplitude, pulse width and interval time, were found significant in both electrolytes. Influences of the pH of electrolyte, balance time, pulse amplitude and pulse width, as well as pH × pH and interval time × interval time were significant in the electrolyte of 0.1 mol/L acetate buffer; electrodeposition time, step increment, pulse amplitude × pulse width and interval time × interval time were significant in the electrolyte of 0.1 mol/L HCl. Their quantitative effects on lead(II) determination were classified into two categories, linear and quadratic, and then quantitative effects were discussed and optimal values were given. Parameters optimization improved the sensitivity of determining trace amounts of lead(II) in solutions by DPASV and alleviated the side effect of dissolved oxygen in the solution with the combination of background subtraction. Through applying Box-Behnken designs for parameters optimization of DPASV in lead(II) determination, peak currents at 20 μg/L, R.S.D. at 20 μg/L and detection limits in both electrolytes were superior than before. With the detection limit of 0.66 μg/L and a precision of 1.08% R.S.D. at the level of 20 μg/L, 0.1 mol/L HCl was a better choice for the kind of electrolytes in this work. It was of great importance and necessity to apply Box-Behnken designs in parameters optimization of DPASV for the analysis of lead(II) no matter what kind of the electrolyte was.

## Materials and Methods

### Apparatus

DPASV was performed in a conventional three-electrode cell with a LK98BII electrochemical workstation (Lanlike Chemical Electronic High Technology Co., Ltd., Tianjin, P.R. China). The working electrode was an Ag-Hg film electrode. An Ag/AgCl electrode (saturated KCl) and a platinum foil were served as the reference and auxiliary electrode. All electrochemical experiments were carried out in a three-compartment electrochemical cell at room temperature (25 ± 1 °C) in the presence of oxygen.

### Reagents and solutions

All chemicals were of analytical grade and bought from Sinopharm Chemical Reagent Co., Ltd, Shanghai, P.R. China. Deionized water (≥18.2 MΩ, MING CHE Water Purification System, Merck Millipore, P.R. China) was applied for the preparation of aqueous solutions. By mixing appropriate amounts of CH_3_COOH and CH_3_COONa, a 0.1 mol/L acetate buffer (HAc-NaAc) was employed as one of the electrolytes. As for the other electrolyte, 0.1 mol/L HCl was performed with the mixture of appropriate amounts of hydrochloric acid and deionized water.

Lead(II) standards with the concentration of 20 μg/L were obtained by diluting the stock solutions (GSB G 62071-90 (8201), National Testing Center of Iron and Steel Materials, Central Iron & Steel Research Institute, P.R. China, 1,000 μg/mL) in both electrolytes.

### Box-Behnken designs

Box-Behnken design, in which significant variables of parameters optimization were indicated, was chosen to investigate linear, quadratic, and cross-product effects of parameters, or factors and presented the corresponding equations that correlate the variables and responses. Crucial factors involved in this work were the pH of electrolyte, electrodeposition potential, termination potential, electrodeposition time, balance time, step increment, pulse amplitude, pulse width and interval time. These nine factors were classified into three categories and each category contained three variables.

In Box-Behnken designs, each variable was varied at three levels and three replicates were conducted in the center, therefore, according to the equation:

$$n=2k(k-1)+C,$$

in which *n* is the number of experiments, *k* is the number of variables (3 for each Box-Behnken design) and *C* is the number of replicates at the center^34^, 15 experiments were carried out for each Box-Behnken design.

Levels of factors were selected based on current researches and details were given in Table 4. The peak current (μA) was applied as the response (*Y*).

**Table 4:** Levels of variables selected for Box-Behnken designs.

Electrolytes	Box-Behnken designs	Variables	Levels		
			−1	0	1
0.1 mol/L acetate buffer (HAc-NaAc)	I	*x*_1_: pH of electrolyte	3.7	4.2	4.7
		*x*_2_: electrodeposition potential (V vs. Ag/AgCl)	−1.2	−0.9	−0.6
		*x*_3_: termination potential (V vs. Ag/AgCl)	−0.2	−0.1	0
	II	*x*_1_: electrodeposition time (s)	60	180	300
		*x*_2_: balance time (s)	10	20	30
		*x*_3_: step increment (V)	0.001	0.003	0.005
	III	*x*_1_: pulse amplitude (V)	0.04	0.06	0.08
		*x*_2_: pulse width (s)	0.05	0.15	0.25
		*x*_3_: interval time (s)	0.1	0.2	0.3
0.1 mol/L HCl	I	*x*_1_: electrodeposition time (s)	60	120	180
		*x*_2_: balance time (s)	10	20	30
		*x*_3_: step increment (V)	0.002	0.004	0.006
	II	*x*_1_: pulse amplitude (V)	0.06	0.10	0.14
		*x*_2_: pulse width (s)	0.6	1.0	1.4
		*x*_3_: interval time (s)	0.05	0.2	0.35

### Measurement procedures

Besides parameters optimized in Box-Behnken designs, sensitivity, filtering parameter and amplification factor in this study were 10 μA, 50 Hz and 1, respectively. Prior to the next determination, the Ag-Hg film electrode was activated for 30 s at 0.3 V to remove the previous deposits completely.

### Data analyses

Data acquired from Box-Behnken designs were analyzed by Design-Expert 10 (Stat-Ease, Inc., USA) and 0.05 was applied as the significant level.
